# How Wealth Accumulation Can Promote Cooperation

**DOI:** 10.1371/journal.pone.0013471

**Published:** 2010-10-27

**Authors:** Thomas Chadefaux, Dirk Helbing

**Affiliations:** ETH Zurich, CLU E1, Chair of Sociology (Modeling and Simulation), Zurich, Switzerland; University of East Piedmont, Italy

## Abstract

Explaining the emergence and stability of cooperation has been a central challenge in biology, economics and sociology. Unfortunately, the mechanisms known to promote it either require elaborate strategies or hold only under restrictive conditions. Here, we report the emergence, survival, and frequent domination of cooperation in a world characterized by selfishness and a strong temptation to defect, when individuals can accumulate wealth. In particular, we study games with local adaptation such as the prisoner's dilemma, to which we add heterogeneity in payoffs. In our model, agents accumulate wealth and invest some of it in their interactions. The larger the investment, the more can potentially be gained or lost, so that present gains affect future payoffs. We find that cooperation survives for a far wider range of parameters than without wealth accumulation and, even more strikingly, that it often dominates defection. This is in stark contrast to the traditional evolutionary prisoner's dilemma in particular, in which cooperation rarely survives and almost never thrives. With the inequality we introduce, on the contrary, cooperators do better than defectors, even without any strategic behavior or exogenously imposed strategies. These results have important consequences for our understanding of the type of social and economic arrangements that are optimal and efficient.

## Introduction

Explaining the emergence and stability of cooperation has been a central challenge in biology, economics and sociology [1]. Unfortunately, the mechanisms known to promote it either require elaborate strategies [2], [3], or hold only under restrictive conditions [4], [5]. More recently, a number of new mechanisms have been shown to facilitate cooperation: the topology of networks determining the interactions among players [6]–[16], even though their robustness has been challenged [17]–[19]; optional participation [20]; or reciprocity [21], [22]. However, even when these conditions are met, cooperation typically merely survives but does not thrive.

Here, we show instead that cooperators can dominate exploiters even without complex strategies and for a wider range of parameters than previous models. We obtain this result by studying heterogeneity among individuals. While this is not the first study to emphasize the importance of diversity on cooperation, we adopt a different approach in which it is neither predetermined nor exogenous. In particular, previous publications have assumed fixed and exogenously imposed heterogeneity in payoffs [23], strategies [24], or the number of interaction partners [25]. Here, on the contrary, we allow a more dynamic and endogenous ‘rich-get-richer effect,’ and we do not impose any strategy on the players. More precisely, we assume that people can accumulate gains, and that this accumulated wealth affects the size of the deals they can potentially reach, so that the rich can get richer [13], [26]. This emergent heterogeneity in which present gains affect future ones is more realistic than the conventional game-theoretical assumption of equal payoffs since, in the real world, the rich typically engage in deals with larger stakes than the poor.

Our study follows the standard literature in analyzing the problem of cooperation by means of ‘games’—simplified mathematical representations of social or strategic dilemmas. In them, people interact in a pairwise fashion with members of their local network, and can take one of two actions: ‘cooperate’ or ‘defect’ (i.e., exploit the other). If both cooperate, they each receive 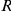; if both defect, they receive 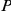; finally, if only one defects and the other cooperates, the exploiter receives 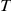, whereas the ‘sucker’ receives 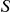. The well-known prisoner's dilemma, for example, is defined by 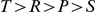. These interactions are repeated over time, and individuals imitate the strategy of the best-performing member of their interaction network (without forecasting).

To this typical setup, we added inequality by varying gains and losses across agents in the following manner. In each interaction, individuals invest a fraction 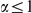 of their wealth (to keep the model simple, we assume a common 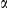 for the entire population), and the return on this investment is then determined by the outcome of the game. Assume for example that an individual with wealth 100 interacts with another with wealth 2. Then, the smaller budget (here: 2) determines the size of the deal and 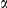 the proportion of wealth actually devoted to it, so that both are assumed to invest 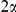. This is intuitive: middle-income individuals cannot enter into multi-million deals, and individuals do not always invest their entire wealth into a single risky deal (hence 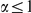).

In turn, the payoffs are logically determined by the size of the deal. To return to our earlier numerical example, if one player cooperates and the other decides to exploit, the cooperator receives a gain of 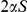, whereas the defector receives 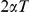. These gains are added to the individual's existing wealth, which in turn affects her future gains. Thus, the nature of the game (be it a prisoner's dilemma, a stag-hunt or a snowdrift game) is preserved, but gains and losses are endogenous, in the sense that they are a function of past performance. This is the main theoretical innovation of this paper: payoffs are not exogenously defined, but rather endogenously determined as a function of the players' past actions.

## Methods

We analyze a spatial game with only two types of behaviors: cooperation (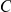) and defection (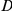). 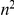 players are randomly assigned an initial strategy (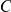 or 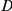) and placed on the sites (cells) of a two-dimensional 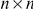 square lattice with periodic boundaries (a torus). Time increases discretely (i.e., we use the standard parallel update, though the results are robust to continuous updating). In each round, every player interacts with each of its four von-Neumann neighbors (denoted by 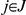) in a pairwise fashion (self-interactions are excluded). Thus, each individual plays four games in each round and her score for the round is the sum of her payoffs in each of these games. The impact of adopting the ‘Moore’ neighborhood instead will be explored below.

Player 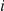
's payoff in her pairwise interaction with player 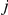 at time 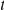 is defined by the matrix
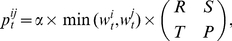
(1)where 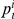 denotes 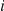
's cumulative payoff at the beginning of round 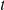. Note that, if we assumed that individuals 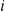 and 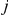 invest different fractions 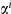 and 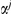 of their wealth, 
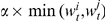
 would just have to be replaced by 
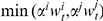
, but this is not a relevant issue in our model. The payoff reflects the idea that the magnitude of the gains (or losses) two players can obtain is limited by the wealth of the weaker player. When a rich person meets a poor one, the stakes of the game they might play are small in absolute terms. Put differently, a rich player cannot force a poor one into potential debt. Finally, to avoid division by zero and the intricacies of negative wealth (‘debt’), we assume that players have a minimal cumulative payoff of 1 (again, this is no crucial model ingredient. Alternatively, all payoffs could be shifted by a constant amount towards positive values.)

At the end of each step, agents update their payoffs and switch their strategy to the one of their most successful neighbor, but do not switch if they were the most successful in that round. By ‘successful’, we mean here the amount of gains obtained during that step. To ensure the robustness of our findings, we also included a mutation mechanism by which at the end of each round, one player is randomly chosen to switch its strategy to defection (no change occurs if a defector is chosen) [27], [28].

Unless otherwise specified, the simulations used to generate the graphs are based on the following setup: 10,000 agents are placed on a 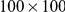 torus, each simulation is run for 1,000 steps, and the results are averaged over 100 different runs for each set of parameters. The default set of parameters is 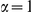, 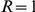, 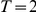, 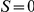, and 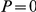 (but 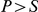 does not change our conclusions). These values are standard in the literature on games, but we also investigated the impact of varying 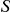 and 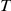 (see below). The interaction network in each simulation is of degree 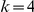, but we have also explored extending it to larger degrees (see results below). We start with 50% cooperators and 50% defectors uniformly distributed in space. The updating rule is synchronous—that is, all agents update their payoff and strategy simultaneously at the end of each step. The asymptotic proportions are determined by averaging over the last 100 rounds of each simulation. In none of the cases is the standard deviation in these last steps large enough to suggest any instability that would warrant a different approach. In other words, the level of cooperation converges to a fixed, parameter-dependent value.

## Results

**Figure 1:**
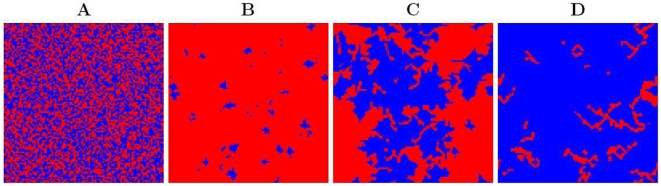
Evolution of cooperation over time. Snapshots of the lattice of a typical run (red denotes defectors, blue cooperators). (A) We start at 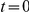 with 50% cooperators. (B) After only a few steps (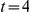), the number of cooperators has dramatically decreased, and only a few cooperative clusters survive. (C) Those who do survive, however, expand (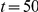) and (D) ultimately take over the entire lattice (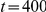).

**Figure 2:**
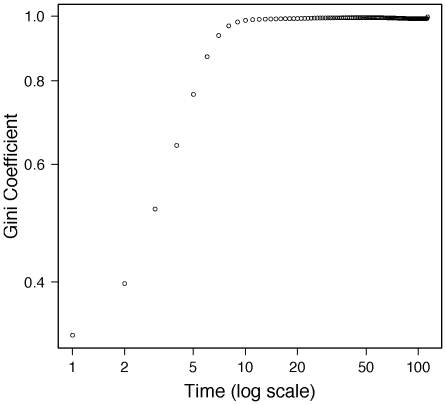
Gini coefficient over time in a typical run. The plot measures inequality by reporting the evolution over time of the population's Gini coefficient. We use the players' cumulative wealth to calculate the coefficient. Because of the setup in which we allow the rich to become increasingly rich, the coefficient rapidly reaches extreme levels, implying that wealth ends up being very unevenly distributed among individuals, with only a few owning most of the total accumulated wealth.

**Figure 3:**
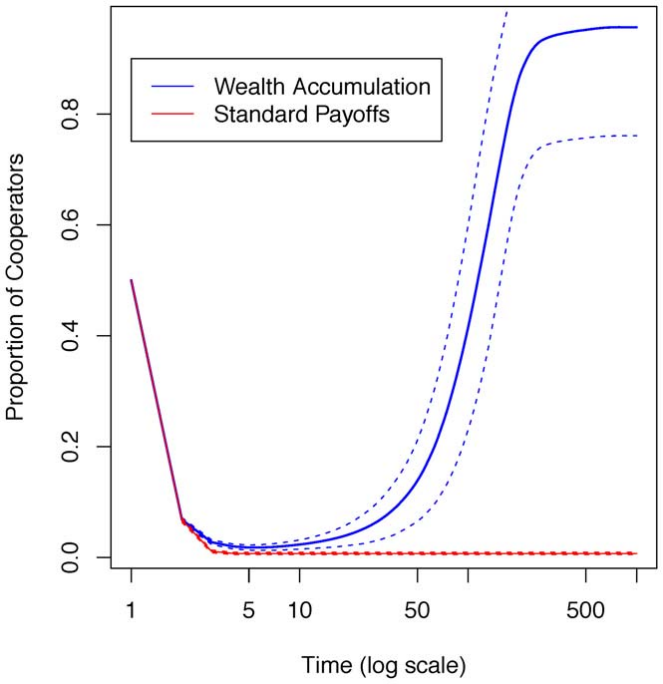
Evolution of the number of cooperators over time. The plot shows the evolution of the number of cooperators over a typical run (see also Figure 1), with (blue line) and without (red line) wealth accumulation. Initially, cooperators perform poorly. However, those cooperators who do survive the initial rounds have accumulated substantial amounts of wealth and are hence able to survive and spread. Over time, the proportion of cooperators converges to 1 (but typically does not reach it). The dashed lines represent two standard deviations.

Extending classical spatial games such as the prisoner's dilemma [29] by wealth accumulation and economic inequality yields striking results. Intuitively, we would expect the ‘rich get richer’ effect to undermine cooperation and, initially, defectors do indeed obtain a high score, while cooperators perform poorly. This is because defectors exploit cooperators, thereby securing an initial level of wealth that allows them to do well. As a result, most players imitate these successful defectors, and cooperation almost disappears from the world (Figure 1B). However, those who do survive are those who were initially isolated from defectors by a cooperative network, and have thereby accumulated a substantial cumulative wealth. They have been at the center of a cluster of cooperators, and hence have been able to accumulate a sizeable wealth in their first rounds. This wealth then makes them ‘competitive’ against defectors (Figure 1C–D). More precisely, their wealth enables them to secure large gains with their peers, but to suffer only small losses when interacting with defectors. This is because defectors, who perform poorly in the defective environment that follows the first rounds, have little to invest, and hence do not pose a great threat to cooperators. Therefore, after the initial turmoil, and as the world becomes more unequal (Figure 2), cooperators rapidly take over the entire lattice and defectors vanish almost completely (Figures 1D and 3).

**Figure 4:**
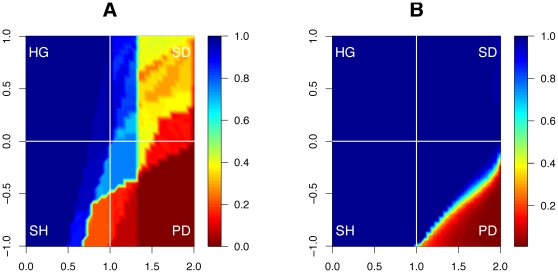
Asymptotic *proportion* of cooperators without and with wealth accumulation. The contour plot shows the average final proportion of cooperators in the world, as a function of the payoff parameters 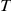 (horizontal axis) and 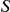 (vertical axis). (A) In a world in which payoffs are homogenous across agents, the proportion of cooperators is low for any 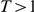. (B) In an unequal environment, in which the rich can become richer, cooperation is stable for a much larger range of payoff parameters. The top-left quadrant corresponds to the harmony game (HG); the bottom-left (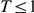 and 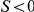) to the stag-hunt (or ‘assurance’) game (SH); the upper-right quadrant (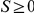, 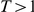) to the snowdrift (or ‘chicken’) game (SD); and the lower-right quadrant (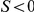 and 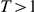) corresponds to the prisoner's dilemma (PD) [33].

These results are in marked contrast to the classical spatial prisoner's dilemma, in which defectors tend to spread and cooperators do not thrive. There are three main ways to appreciate these differences. First, in an equal world (the classical model) in which payoffs are homogenous across agents, the final proportion of cooperators is low for a wide range of parameters—in particular those that define the prisoner's dilemma. That is, it is very rare and difficult to obtain cooperation, and even more difficult to sustain it without the rich-get-richer dynamics considered here. In our setup, however, cooperation is stable for a much larger range of parameters. More precisely, the asymptotic *proportion of cooperators* is larger for a wide range of parameter values (Figure 4), including in the prisoner's dilemma and the snowdrift (or ‘chicken’) games.

**Figure 5:**
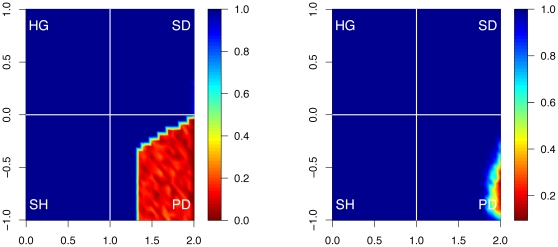
*Survival* of cooperation without and with wealth accumulation. The contour plot shows the percentage of runs in which at least 1% of cooperators survive after 1000 steps, as a function of the payoff parameters 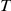 (horizontal axis) and 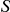 (vertical axis). Note that cooperation can survive in much more hostile conditions (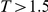 and 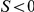) when payoffs are unequal (panel B) than when they are not (panel A). In particular, for extreme values (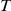 close to 2 and 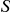 close to −1), cooperation never survives without wealth accumulation, but can survive with it.

Second, cooperation *survives* more often in our setup. In fact, cooperation fails to survive only very rarely and only for the most extreme payoff parameters (Figure 5). This is in particular contrast the conventional prisoner's dilemma (without wealth accumulation).

**Figure 6:**
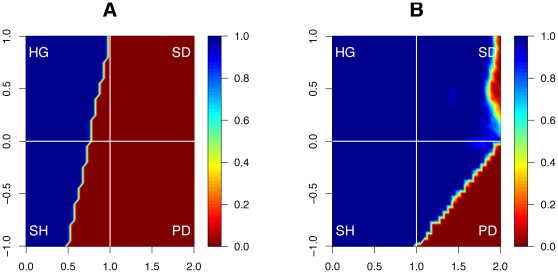
*Domination* of cooperation without and with wealth accumulation. The contour plot shows the percentage of runs that end with more than 99% cooperators, as a function of the payoff parameters 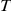 (horizontal axis) and 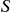 (vertical axis). In a world in which wealth is not accumulated (A), the range of parameters for which cooperation can take over is very limited (blue area in A). When the rich get richer (B), however, the range of parameters is far larger (blue area in B).

Finally, and even more strikingly, we find that cooperators *dominate* defectors for a far wider range of parameters than under the classical game rules. That is, not only can cooperation survive more often, but it thrives and dominates far more than in an equal world (Figure 6). Without wealth accumulation, the domination of cooperators is rare for most parameter values—and in particular for the notoriously hostile parameters that define the prisoner's dilemma and the snowdrift games. With wealth accumulation, however, cooperators fail to dominate defectors only for extreme values of the prisoner's dilemma.

**Figure 7:**
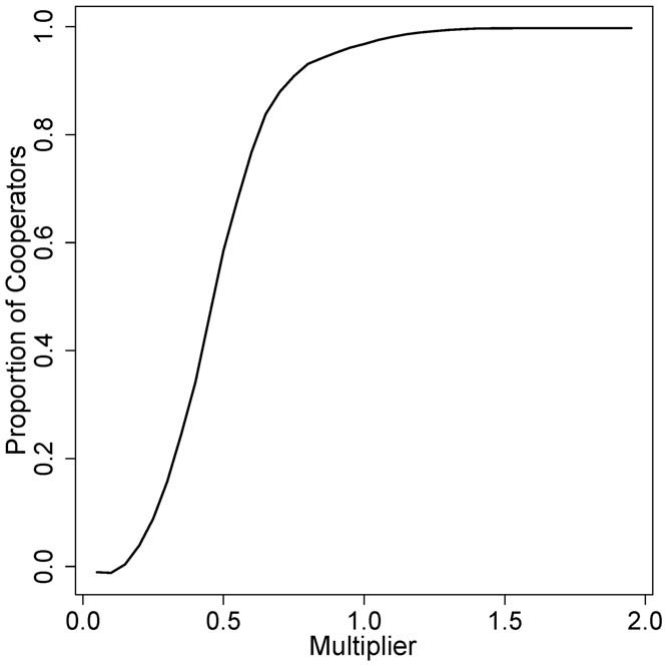
Final proportion of cooperators as a function of 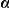. This plot shows the impact of the multiplier 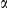 (see eqn. 1) on the final average proportion of cooperators in the world, for typical parameter values.

**Figure 8:**
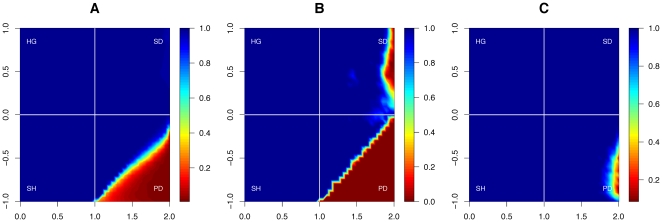
Asymptotic proportion, domination, and survival of cooperators, when individuals imitate others based on cumulative wealth. We tested the robustness of our results by using cumulative payoffs instead of the current step's payoff as the basis of adaptation. That is, agents here adapt to the strategy of their most successful neighbor, as measured by the total wealth they have accumulated over time, instead of the payoff they obtained in the previous step. Panel A shows the final proportion of cooperators in the world (compare with Figure 4). Panel B shows the percentage of runs that end with more than 99% cooperators (compare with Figure 6). Panel C shows the percentage of runs in which at least 1% of cooperators survive after 1000 steps (compare with Figure 5). Note that the results basically agree with the ones, when individuals imitate others based on their payoff in the previous time step, rather than their overall wealth, as is the case here (see Figures 4B, 5B and 6B).

We also investigated the impact of varying the proportion of wealth that individuals invest (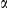). This proportion determines how much past gains affect present benefits, i.e. the rate of wealth accumulation. 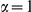, for example, means that present payoffs depend on the full amount of past benefits. 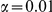, on the other hand, means that only a small portion of the accumulated wealth affects present payoffs and, correspondingly, the rich-get-richer effect is small. We find that the larger the “rich-get-richer effect” (Figure 7), the more likely cooperation is to prevail. However, there is no noteworthy increase in the level of cooperation beyond a value of 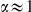. Furthermore, we tested the impact of using total, cumulative payoffs over time instead of relying on the previous step's payoff only, and found our results to be robust to this specification (Figure 8).

**Figure 9:**
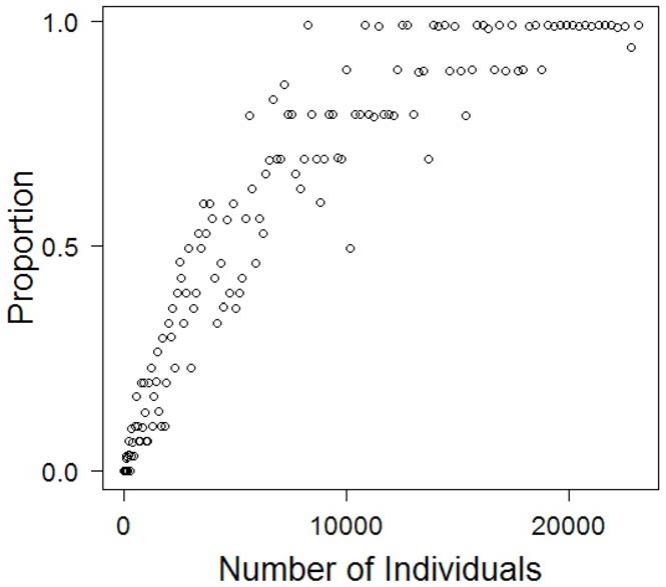
Cooperation as a function of the number of individuals. Asymptotic proportion of cooperators as a function of the number of individuals. A larger number of individuals increases the likelihood that an initial supercritical cluster of cooperators will survive, and hence that at least one cooperator is protected sufficiently long to accumulate enough wealth to outcompete defectors. Hence, for any given set of parameters, a large number of individuals increases the probability with which cooperation will prevail.

We also investigated the robustness of our findings to changes in the number of individuals in the world, and found a strong positive correlation with the likelihood that cooperation emerges (Figure 9). That is, for any payoff parameters, adding individuals on the lattice increases the probability that cooperation will survive and dominate. The logic behind this result is that the survival of cooperators during the first steps of the game is critical. Large worlds—those with many individuals—are likely to have at least one cluster of cooperators of sufficient size to survive the initial turmoil. Since one such cluster is sufficient to foster and promote the eventual spread of cooperation, a large world also increases the chances that cooperation eventually spreads.

**Figure 10:**
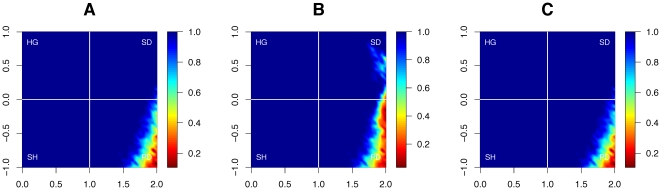
Asymptotic proportion, domination, and survival of cooperators with wealth accumulation under the Moore neighborhood. The contour plots shows three different statistics as a function of the payoff parameters 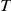 (horizontal axis) and 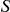 (vertical axis), when the players' interaction network is of degree 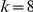 (‘Moore’ neighborhood). Panel A shows the final proportion of cooperators in the world (compare with Figure 4). Panel B shows the percentage of runs that end with more than 99% cooperators (compare with Figure 6). Panel C shows the percentage of runs in which at least 1% of cooperators survive after 1000 steps (compare with Figure 5). We note in particular that the results we obtained with the von-Neumann neighborhood hold under the Moore neighborhood, and even that the performance of cooperators is often improved as a result.

Finally, we changed the degree of the players interaction network from 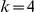 (von-Neumann neighborhood) to 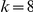 (Moore neighborhood). Not only do our results generalize to this extended neighborhood, but they are even reinforced by it (Figure 10). The underlying reason is the following: when the interaction network is large, the likelihood that an initial supercluster of cooperators emerges is low, since it requires a large set of contiguous players with a cooperative strategy—the likelihood of which decreases as the interaction network increases, since a cluster of 8 surrounding cooperators is far less likely than one of 4. However, any such cluster is also much stronger and resistant to invasion than only of 4 players. In other words, the player at the center of this protective cluster is able to play cooperate over more interactions at the beginning of the game than the player surrounded by only 4 cooperators. As a result, the Moore neighborhood leads to the emergence of very strong cooperative players that are able to sustain the initial cluster and to invade the rest of the world. Of course, the principle is transferable to larger neighborhoods than 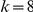, but unfortunately the probability that a protective cluster emerges becomes vanishingly small as 
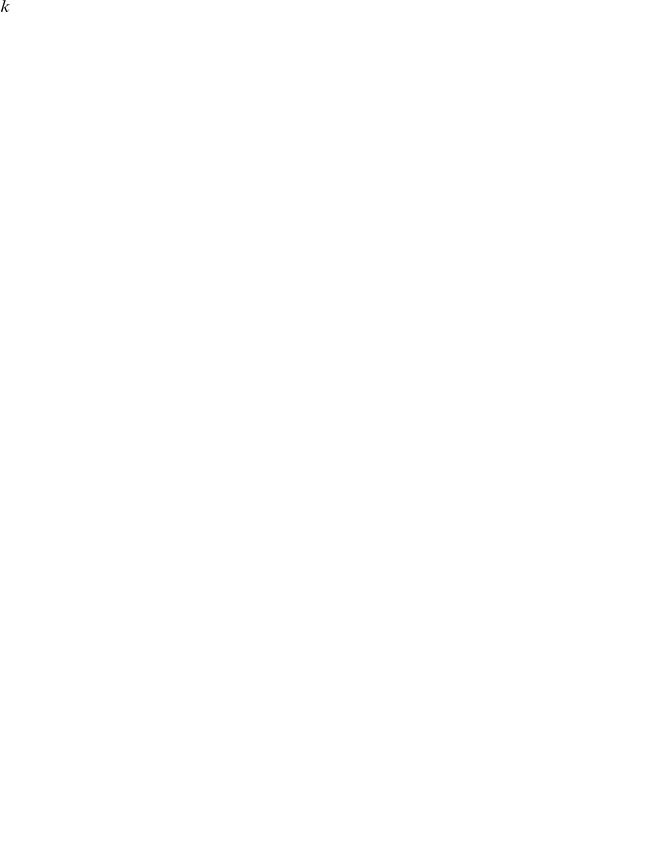
 increases. Hence, cooperation then only emerges when the world becomes sufficiently large to ensure (probabilistically at least) the initial appearance of this cluster.

## Discussion

Our results highlight that emergent heterogeneity through the rich-get-richer effect can support the welfare of society. Some economic inequality can dramatically and unexpectedly promote cooperation, far beyond what would be possible without it. However, this should not be construed as a call for higher levels of inequality, since the marginal effect of inequality is decreasing. Rather, it is the ability for some to build upon previous success that has an impact on cooperation, but increasing inequality itself might not be helpful, and could even become detrimental. Nevertheless, this result has broad theoretical and practical implications.

At the theoretical level, this finding adds to the existing and growing research on the impact of diversity on cooperation [23]–[25], and more generally on evolutionary games on graphs [30]. In particular, it shows how cooperation can be reached in highly hostile environments without any strategic behavior or memory, and for a wide range of parameters and modeling choices. It also points to important future avenues for research that could be explored. For example, the amount of wealth invested in each round could be a parameter determined evolutionarily and specific to each individual. It could also be conditional upon the opponent's strategy: small investments would be made against probable defectors, whereas large ones would be reserved for cooperators. In turn, this might create incentives to defect at the highest level, when the stakes have become large. Another extension would be to allow voluntary contributions across individuals. Donations by the rich to their neighbors (or perhaps strategically to specific key individuals or areas of the world) could help foster more cooperation faster by ensuring the survival of a protective group around them. In this sense, inequality is not the key to cooperation. Rather, the ability of a few to become richer and to reward those who cooperate would be a powerful mechanism to prevent the spread of defection. Finally, more work is needed on including more complex strategies into the present framework. How, for example, does tit-for-tat perform in this context? Are there long-term strategies that would first establish a reputation for cooperation, and then use their dominating position to exploit others? In other words, is the high level of cooperation obtained in our framework susceptible to exploitation by more complex and long-term strategies?

At a practical level, our result goes against our intuition that inequality and conflict (e.g., civil war or class tensions) are positively correlated. However, at least for a moderate inequality, there is little empirical evidence linking the two, whether macro-economic data on civil wars [31] or micro-behavioral data from experiments [32] is used. The US, for example, despite its high economic inequality, is relatively exempt from class conflicts.

Moreover, the recent financial crisis has raised fundamental questions regarding appropriate incentive structures and income distributions. In particular, the extreme incomes in the financial sector have caused much debate and concern. While we do not pretend that our study can offer practical recommendation regarding this issue, we note that the ability to accumulate wealth is key to the success of cooperation in our model. However, this does not mean that the extreme inequality observed between Wall Street and “Main Street” is desirable or even beneficial at all. It might well be that, combined with other factors, growing inequality leads to more conflict rather than cooperation. In addition, while the spread of incomes could in principle be beneficial if it resulted from cooperative behavior (since it would then promote the spreading of cooperation by imitation), it is likely to promote a temporary spreading of defection if it is based on exploitation. Hence, we wish to warn against applying our findings too literally for policy purposes before additional work on more complex strategies has been conducted.
